# Mitochondrial Transport and Turnover in the Pathogenesis of Amyotrophic Lateral Sclerosis

**DOI:** 10.3390/biology8020036

**Published:** 2019-05-11

**Authors:** Veronica Granatiero, Giovanni Manfredi

**Affiliations:** Feil Family Brain and Mind Research Institute, Weill Cornell Medicine, 407 East 61st Street, New York, NY 10065, USA; veg2003@med.cornell.edu

**Keywords:** mitochondria, ALS, axonal transport, mitophagy, SOD1, Miro1, PINK1, Parkin

## Abstract

Neurons are high-energy consuming cells, heavily dependent on mitochondria for ATP generation and calcium buffering. These mitochondrial functions are particularly critical at specific cellular sites, where ionic currents impose a large energetic burden, such as at synapses. The highly polarized nature of neurons, with extremely large axoplasm relative to the cell body, requires mitochondria to be efficiently transported along microtubules to reach distant sites. Furthermore, neurons are post-mitotic cells that need to maintain pools of healthy mitochondria throughout their lifespan. Hence, mitochondrial transport and turnover are essential processes for neuronal survival and function. In neurodegenerative diseases, the maintenance of a healthy mitochondrial network is often compromised. Numerous lines of evidence indicate that mitochondrial impairment contributes to neuronal demise in a variety of neurodegenerative diseases, including amyotrophic lateral sclerosis (ALS), where degeneration of motor neurons causes a fatal muscle paralysis. Dysfunctional mitochondria accumulate in motor neurons affected by genetic or sporadic forms of ALS, strongly suggesting that the inability to maintain a healthy pool of mitochondria plays a pathophysiological role in the disease. This article critically reviews current hypotheses on mitochondrial involvement in the pathogenesis of ALS, focusing on the alterations of mitochondrial axonal transport and turnover in motor neurons.

## 1. Introduction 

Amyotrophic lateral sclerosis (ALS) is a devastating neurodegenerative disorder that causes the death of both upper and lower motor neurons. It is the most common among the motor neuron diseases. Loss of motor neurons results in muscle denervation leading to progressive muscle weakness, causing respiratory failure, difficulty in speaking and swallowing, and eventually paralysis and death, typically between one and five years from the time of disease onset [1]. There are currently no effective treatments for ALS, with only two food and drug administration (FDA) approved drugs, which only extend survival by a few months [2]. Approximately, 10% of ALS cases are due to genetic causes, the remaining 90% are sporadic with unknown etiology [3]. The last decade has brought tremendous advances in the understanding of ALS genetics with over 20 different genes identified that account for almost 80% of all familial forms [4].

The nature of the genes involved in ALS indicates that this is an etiologically heterogeneous disease [5] with a multiplicity of initiating factors that trigger diverse pathogenic pathways, ultimately converging in motor neuron toxicity. In the majority of genetic forms of ALS, motor neuron degeneration is accompanied by the involvement of other neural systems, frequently resulting in frontotemporal dementia (FTD). Some of the most prominent alterations observed in ALS/FTD involve protein homeostasis, autophagy, RNA metabolism, axonal transport and mitochondrial abnormalities [5,6,7,8,9,10,11,12].

Mitochondrial abnormalities have often been implicated as secondary mechanisms of disease, because until recently no genetic forms of ALS have been ascribed to mutations in nuclear or mitochondrial DNA encoded proteins. Only in 2014, the first mutation in a mitochondrial gene, coiled–coiled helix containing domain 10 have been identified in pedigrees presenting with ALS, FTD, and myopathy [13]. Despite the scarcity of primary mitochondrial forms of ALS, mitochondrial alterations can be caused by mutant proteins that are not exclusively localized in mitochondria. Notable examples are mutations in Cu, Zn superoxide dismutase (SOD1), the first genetic form of ALS identified [14], and TAR DNA-binding protein (TDP43, [15]). 

While motor neurons are the most affected cells in ALS, studies on the pathophysiology of the disease have highlighted the importance of non-cell autonomous mechanisms, which implicate other cell types in the central nervous system. Glial cells, including astrocytes [16,17,18], oligodendrocytes [19], and microglia [20,21] play toxic roles in mutant SOD1 mouse models of ALS. Particularly in astrocytes, mitochondria can play a significant role in causing toxicity to motor neurons. For example, failure of astrocytes to clear synaptic glutamate as a result of altered mitochondrial glutamate intermediary metabolism, can trigger neuronal excitotoxicity.

Importantly, in ALS neurons mitochondrial alterations are accompanied by defects of organelle dynamics, involving fusion, fission, and transport [12]. These impairments result not only in morphologically and functionally defective mitochondria, but also in mislocalized mitochondrial network [22]. In excitable cells with large axoplasm, such as motor neurons, loss of viable mitochondria at sites of high energy demand, such as the synapses that motor neurons form with muscle (neuromuscular junctions, NMJ), can have catastrophic consequences leading to muscle denervation. 

This review article focuses on the alterations of mitochondrial transport in neurons and the role of mitochondrial quality control mechanisms in ALS.

## 2. Mitochondrial Axonal Transport in ALS

### 2.1. The Mitochondrial Transport Machinery

In neurons, mitochondria are highly dynamic organelles. They are characterized by fast transport along neuronal axons, in both anterograde and retrograde directions (i.e., from the soma to the periphery and vice versa). Mitochondria from the soma are anterogradely transported to sites where metabolic demand is high, such as the synapse [23,24], while retrograde transport provides essential information regarding the status and environment of distal sites. Thus, mitochondrial transport plays an essential role in maintaining healthy motor neurons, and alterations of rate-limiting components of the mitochondrial transport machinery may cause an imbalance of mitochondrial distribution in neurons. Furthermore, as active lysosomes are mostly localized in the soma, damaged mitochondria in axons are engulfed in autophagosomes and retro-transported to the soma to be degraded upon fusion with lysosomes [25]. Defective retrograde transport of autophagosomes could result in a delay of mitochondrial autophagy (mitophagy) fluxes and accumulation of damaged mitochondria. Thus, axonal transport and mitophagy are intimately interconnected processes. 

For long-range axonal transport mitochondria move along microtubules. The kinesin superfamily of proteins and cytoplasmic dynein are the main microtubule-based motor proteins. They drive long distance transport of mitochondria and other membranous organelles through ATP-dependent mechanisms [26]. In axons, cytoplasmic dynein is responsible for the retrograde transport, moving mitochondria towards the soma, whereas kinesin drives anterograde mitochondrial transport from the soma to distal axonal regions and synaptic terminals. In dendritic spines, where microtubules exhibit mixed polarity in proximal regions, kinesin and dynein motors can transport mitochondria in either direction, depending on the microtubule polarity [26,27]. There are two main mechanisms by which molecular motors connect with their cargoes, direct linkage through cargo motor proteins or indirect linkage via linker/adaptor molecules. Several adaptor complexes have been identified, which ensure a precise regulation of mitochondrial motility. Among them, the best studied linkers are Milton and Miro, first identified in Drosophila. Milton is a kinesin heavy chain-binding protein. In mammals, there are two Milton orthologues, TRAK1 and 2. Milton is linked indirectly with the mitochondrial outer membrane through interaction with an atypical Rho GTPase, Miro. Mammals have 2 orthologues of Miro (Miro1 and 2) [28,29]. Together Miro, Milton and kinesin provide mitochondria-specific axonal transport mechanisms. 

The ATP/ADP ratio is part of the signaling involved in regulating mitochondrial transport. In regions with high ATP level, mitochondrial velocity increases, while upon ATP depletion mitochondrial velocity decreases [30]. Another important signal is provided by neuronal Ca^2+^ concentration. Miro contains two Ca^2+^-sensitive helix-loop-helix structural domain, also called EF hand motifs, facing the cytosolic side, which sense cytosolic Ca^2+^ levels. Two different mechanisms have been proposed to explain how Miro regulates mitochondrial motility in a Ca^2+^-dependent manner. According to the “motor-Miro binding” model, when Ca^2+^ around mitochondria is low, the C-terminal tail of kinesin is bound to the mitochondrion through its interaction with the Milton–Miro complex, thereby allowing for mitochondrial transport. When Ca^2+^ is high, it binds to Miro EF hands, causing a conformational change that results in the direct interaction of kinesin with Miro, which prevents mitochondrial movement [31]. According to the “motor-releasing model”, upon Ca^2+^ increase, Miro remains attached to Milton and the mitochondrion, but dissociates from the kinesin, thereby arresting mitochondrial transport [32].

### 2.2. Alterations of the Mitochondrial Transport Machinery in ALS

There are two main hypotheses on the process of motor neuron degeneration in ALS, the “dying-forward” and the “dying-back” hypotheses. The former proposes that ALS is mainly a cortical motor neuron disorder, which mediates anterograde degeneration of anterior horn cells via glutamate excitotoxicity [33]. On the other hand, the “dying-back” hypothesis proposes that motor neurons degeneration in ALS starts distally at the nerve terminal or at the NMJ and progresses towards the soma [34]. In support to the latter, it was shown that early degeneration of the NMJ precedes the loss of neurons in the spinal cord of mutant SOD1 mice [35,36]. Mitochondrial transport abnormalities could significantly contribute to dying-back processes, because the distal regions of motor neurons may not be appropriately supplied with healthy, functional mitochondria, while damaged mitochondria may not be correctly turned over. A significant body of evidence points towards a causal relationship between deficits in axonal transport and degeneration of susceptible motor neurons in ALS [37,38]. For example, defects in neuronal mitochondrial morphology and axonal mitochondrial transport have been demonstrated in primary neuronal cultures from ALS mouse models [12,39,40]. Importantly, these abnormalities have also been observed in vivo in mutant SOD1 and TDP-43 ALS mouse models [41,42] and in Drosophila models [43]. Other studies in primary neuronal cultures and in vivo demonstrated that mutant FUS, a RNA-binding protein causative of familial ALS/FTD [44], induces motor neuron degeneration preceded by abnormalities in synaptic transmission [45] and mitochondrial abnormalities at the NMJ [46]. The common denominator of these studies was the finding that mitochondrial abnormalities and the impairment of mitochondrial axonal transport precede motor neuron degeneration. This evidence supports the “dying-back” hypothesis [34,47,48], in which mitochondrial transport abnormalities may play an instrumental role. Indeed, in mutant SOD1 mice deficits in bidirectional transport of mitochondria are described at the pre-symptomatic disease stage [49], suggesting that these alterations play an early causative role in NMJ degeneration.

The causes of mitochondrial transport abnormalities in ALS motor neurons are not fully understood. In some cases, alterations of the axonal transport machinery are directly involved. For example, studies suggest that SOD1 mutations impair dynein functions, as mutant SOD1 directly interacts with the dynein–dynactin complex, forming aggregates in the spinal cord and sciatic nerve of SOD1 transgenic mice [50,51]. Furthermore, a large body of evidence in models of familial ALS with mutations in SOD1 or TDP-43 indicates that mitochondrial damage and dysfunction is the result of the pathological accumulation of aggregated mutant proteins inside or on the surface of mitochondria [42,52,53,54,55,56,57,58]. Therefore, another possibility is that ALS mutant proteins damage mitochondria and impair their bioenergetics [59,60,61], thereby decreasing ATP availability for axonal transport. This is an attractive hypothesis, because it could provide a mechanism whereby unhealthy mitochondria are immobilized to facilitate their removal by autophagy, similar to the Ca^2+^ dependent mitochondrial arrest described above [31,32]. While it was shown that ATP levels affect mitochondrial motility [30], to our knowledge, the question of whether subpopulations of energy defective mitochondria have a selective decrease in transport, has not been addressed experimentally. This could be achieved, for example, by causing a mild impairment in ATP synthesis in a targeted subset of mitochondria in the neuronal soma and tracking their movement to neuronal processes over time, in comparison with healthy mitochondria.

Although long-range mitochondrial transport is microtubule-based, short range movement in presynaptic terminals and dendritic spines, where actin filaments form the cytoskeletal architecture, is mediated by actin-myosin motors. In cultured neurons, axonal mitochondria have been shown to travel along microtubules and actin microfilaments with different velocities and mechanisms [62]. The actin cytoskeleton is especially relevant to motor neuron diseases with altered actin dynamics [63]. Profilin1 (PFN1) is one of four isoforms of profilin, and the first actin-binding protein associated with familial ALS [64]. Initial studies suggested that the main function of profilin was to sequester actin monomers, thereby inhibiting F-actin formation [65,66]. However, later studies revealed that the amount of profilin present in cells is not sufficient to sequester abundant actin monomers, and a different function for profilin was proposed, as a catalytic converter for actin monomer recycling [67,68]. There are several hypotheses on the pathogenic mechanisms of mutant PFN1 in ALS. Both gain and loss of function have been proposed. The former is based on mutant PFN1 forming aggregates in motor neurons [69,70]. The latter on studies showing that mutant PFN1 causes the formation of cavities in its protein core structure, compromising protein stability and leading to misfolding and degradation [71]. Motor neurons of mutant PFN1 transgenic mice show aggregation of the protein and disruption of the actin cytoskeleton, accompanied by elevated ubiquitin and p62/SQSTM levels in motor neurons [72]. Another transgenic mouse model of mutant PFN1 revealed alterations of actin dynamics and reduced filamentous versus globular actin ratio [73]. Although mitochondrial transport and turnover have not yet been investigated in these mouse models, alterations in mitochondrial ultrastructure was reported in motor neuron axons [73], suggesting that cytoskeletal alterations in these mice affect mitochondria, possibly through impairment of their dynamics.

Myosins allow for cargo movement along actin cytoskeleton. Myo19 is the only myosin localized to mitochondria, and plays a physiological role in mitochondrial movement under conditions of glucose-starvation [74]. Recently, a new intersection point between microtubule-dependent and actin-dependent mitochondrial movement was described through Miro [75,76]. It was shown that, upon activation of pathways of mitochondrial degradation, Myo19 was digested together with Miro, thereby regulating mitochondrial movement and distribution. These findings raise the possibility that detachment of mitochondria from the actin cytoskeleton may be an important step in altering mitochondrial transport. Together these findings strongly suggest that microtubule- and actin-dependent mitochondrial transport mechanisms may be connected and that both mechanisms could be dysregulated in ALS.

## 3. Mitochondrial Turnover in ALS

### 3.1. Mitochondrial Quality Control Mechanisms

Mitochondrial quality control (MQC) is an important process in cellular homeostasis. Mitochondria with loss of membrane potential or subject to protein oxidation and misfolding become targets of MQC. There are three main known pathways of MQC: Protein degradation, vesicular degradation, and mitophagy. The first involves proteostatic selective elimination of damaged proteins. Mitochondria have internal proteases, such as the AAA-protease complex of the inner membrane [77] and the Lon protease of the matrix [78]. Mitochondria are also endowed with their own unfolded protein response, which is activated when misfolded proteins accumulate in the matrix [79] or in the intermembrane space [80]. Mitochondria rely on the cytosolic ubiquitin-proteasome system to eliminate damaged proteins destined to the outer membrane or in the case of the intermembrane space, before they engage in the mitochondrial import pathway [81,82]. Ubiquitin-ligases, such as Parkin, ubiquitinate oxidized or misfolded outer membrane proteins [83]. Parkin recruitment has been ascribed to the kinase PINK1, following its incomplete processing and import across the outer membrane of depolarized mitochondria [84,85,86]. Instead, maintenance of Parkin cytosolic localization has been attributed to its interaction with cleaved PINK1 released in the cytosol after processing by healthy mitochondria [87]. If its degradation fails in damaged mitochondria, PINK1 phosphorylates ubiquitin residues and Parkin, thereby activating a cascade of ubiquitination of outer membrane proteins [88]. Ubiquitination of outer membrane proteins is not exclusively performed by Parkin, as it can also be carried out by resident outer membrane ligases, such as MULAN [89] and MITOL/MARCH5, which ubiquitinate outer membrane proteins involved in mitochondrial fusion, Mfn1 and Mfn2, and fission, Drp1 [90,91]. Interestingly, MARCH5 ubiquitinates and increases the turnover of mutant SOD1 on the outer membrane [92].

Ubiquitination of outer membrane proteins is one of the best characterized signals for the activation of mitophagy and the regulation of mitochondrial motility through degradation of proteins involved in mitochondrial fusion/fission and transport (reviewed in [93]). Fragmentation of the network and immobilization facilitate the engulfment of damaged mitochondria in autophagic vesicles. The regulation of PINK1 by phosphorylation of specific amino acid residues [94] could provide an additional link between energetic defects in unhealthy mitochondria and the activation of MQC. What drives the switch from proteostasis to mitophagy is unclear, but the extent of mitochondrial damage is likely a discriminating factor: When proteostasis cannot repair mitochondria, mitophagy ensues. However, in some cases, a Parkin-dependent vesicular degradation of sections of mitochondrial membranes containing oxidized proteins, can be sufficient to repair the damage and prevent full-blown mitophagy [95]. Mitophagy involves ubiquitin-binding adaptors that recruit mitochondria to the autophagosome by binding to LC3 [88].

### 3.2. Mitochondrial Quality Control in ALS

The best characterized MQC pathway is mediated by the activation of PINK1 and Parkin [96]. Impairment of MQC is most commonly linked to Parkinson’s disease, due to the discovery of inactivating mutations of these proteins in recessive forms of the disease [97,98,99]. However, MQC disturbances have also been associated with ALS, for example through the involvement of the mitophagy adaptor optineurin, which has been found to be mutated in familial forms of the disease [100]. Optineurin plays a role in PINK1-Parkin mediated mitophagy. After Parkin recruitment to the outer membrane, optineurin binds to ubiquitinated mitochondria, inducing autophagosome nucleation through LC3 recruitment [101]. ALS-associated optineurin mutations cause mitochondrial clearance impairment [102]. The link between optineurin and LC3, which finalizes autophagic clearance of damaged mitochondria, is Tank-binding kinase (TBK1). It was reported that TBK1 interacts with optineurin and by phosphorylating it at specific serine residues regulates its ability to bind to ubiquitinated mitochondrial proteins [103]. Therefore, TBK1-mediated phosphorylation of optineurin amplifies and reinforces mitophagy. As a consequence, inhibition or depletion of TBK1 delays mitophagy, resulting in accumulation of damaged mitochondria [104]. TBK1 mutations have been causally linked to familial forms of ALS [105], further reinforcing the relevance of MQC in ALS pathophysiology. Mutations in two additional genes involved in mitophagy were reported in familial ALS, p62 and VCP. p62/SQSTMQ1 is an adaptor for the binding of ubiquitin to LC3, recruiting mitochondria to the autophagosome [106,107]. Valosin-containing protein (VCP) is an ATPase with segregase activity, which can extract ubiquitinated proteins from organelle membranes and target them to proteasomal degradation [108]. Parkin-mediated outer membrane protein ubiquitination recruit VCP to mitochondria [109]. Mutations in VCP are associated with ALS [110]. VCP mutations cause mitochondrial structural changes in transgenic mice, and loss of VCP impairs the clearance of damaged mitochondria [109]. Overall, the genetics of familial ALS strongly emphasizes the relevance of MQC in disease pathogenesis. 

Mitochondrial damage with loss of mitochondrial membrane potential may result in Parkin-mediated degradation of Miro [111]. Furthermore, it was reported that PINK1 directly interacts with Miro and Milton [112], and that it phosphorylates Miro in response to mitochondrial damage, thereby promoting its interaction with Parkin and its degradation [113]. Interestingly, Miro is decreased in ALS models [114,115], which may contribute to impairing mitochondrial axonal transport. Moreover, Miro overexpression in mutant SOD1 neurons restores mitochondrial axonal transport deficits [116]. Taken together, these data point to a role for Miro as a converging point between the mechanisms regulating MQC and axonal transport.

The actin cytoskeleton emerges as an additional converging point between mitochondrial turnover and mitochondrial motility. A recent study showed that, after Parkin recruitment to depolarized mitochondria, F-actin encapsulates damaged mitochondria through myosin VI (MYO6) complexing with Parkin [117]. Hence, damaged mitochondria are completely isolated from the rest of the mitochondrial network, and are prevented from fusing with healthy mitochondria. They also showed that lack of MYO6 induces accumulation of damaged mitochondria, because of a severe impairment in the clearance machinery, highlighting the importance of actin-related players in MQC and maintenance of mitochondrial homeostasis.

The therapeutic value of modulating autophagy and mitophagy in ALS is the object of debate. Stimulation of autophagy was attempted mostly in the SOD1 mouse model, using both pharmacological and genetic approaches [118,119,120,121,122,123]. There were discrepancies in the outcomes, with either beneficial or detrimental effects. This divergence of results could derive from the different approaches used and their effects on different central nervous system (CNS) cell types. A genetic approach to test the cell type specificity of autophagy modulation was implemented by deleting the critical autophagy gene Atg7 specifically in motor neurons of mutant SOD1 mice [124]. Autophagy inhibition accelerated early neuromuscular denervation and the onset of motor symptoms. Surprisingly, removal of Atg7 also extended the lifespan of the animals. The authors proposed that motor neuron autophagy contributes to maintaining neuromuscular innervation early on, but later causes a non-cell-autonomous effect that promotes disease progression. Although mitophagy was not investigated specifically, the findings raise the intriguing possibility of a phase-dependent involvement of MQC in ALS progression.

Mitophagy induction in ALS is supported by the finding of LC3 II increase in neurons of mutant SOD1 mice [125,126], accompanied by the accumulation of p62 and optineurin [115,127]. Furthermore, an increase in mitochondria-containing autophagosomes and autophagolysosomes was described in human ALS spinal cord [128]. Currently, there are no pharmaceutical approaches to selectively modulate MQC in ALS. However, it is possible to target genetically the main components of the machinery, such as Parkin. Interestingly, a progressive decrease in Parkin levels was documented in both cellular and animal models of ALS [115,129,130], suggesting that chronic activation of MQC secondary to mitochondrial damage causes Parkin depletion. In cultured ALS neurons, it was demonstrated that Parkin is responsible for Miro degradation and actively contributes to mitochondrial transport impairment [116]. Overexpression of Miro or ablation of PINK1 rescued the mitochondrial axonal transport deficits. However, genetic constitutive ablation of Parkin in mutant SOD1 mice delayed the decline of Miro and other components of the mitochondrial dynamics machinery, and resulted in a significant delay of neuromuscular degeneration and extension of lifespan [115]. Taken together, these findings support the notion that MQC is involved in ALS pathophysiology. Importantly, they also show that autophagy, and specifically MQC, may be a double-edge sword, with initial protective effects, which can become maladaptive and detrimental in the chronic phase of the disease. 

## 4. Conclusions

**Figure 1:**
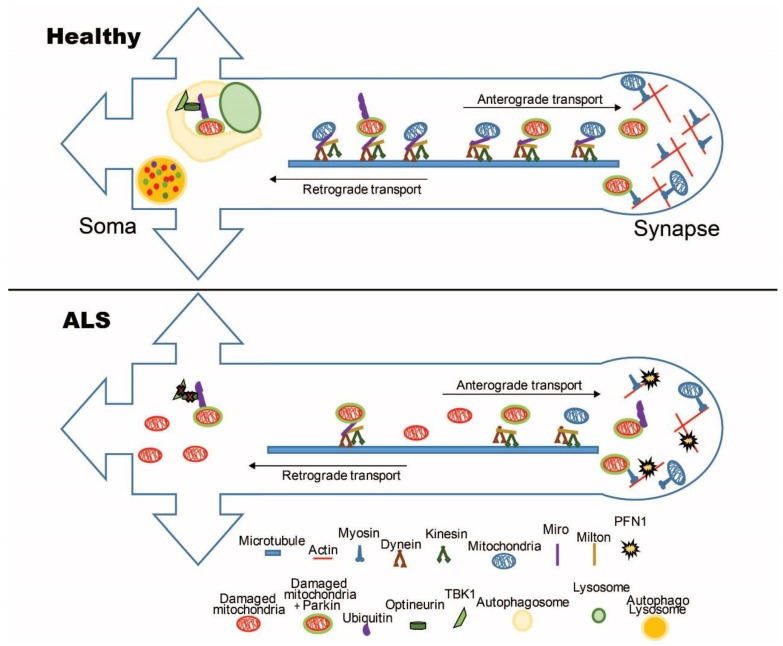
Schematic representation of key players in mitochondrial transport and turnover in healthy and amyotrophic lateral sclerosis (ALS) neurons. Mitochondria are transported in axons along microtubule tracks by dynein and kinesins, which are connected to mitochondria through cargo adaptors Milton and Miro. In ALS axons, the interactions between mitochondria and microtubules are disrupted, resulting in impaired transport. At synapses, mitochondria interact with the actin cytoskeleton, and mutations in proteins involved in actin dynamics, such as PFN1, can alter mitochondrial localization at this neuronal site. Ubiquitination of unhealthy mitochondria by Ub-ligases, such as Parkin, target mitochondria for degradation through the autophagy pathway. TBK1 and optineurin promote PINK1-Parkin ubiquitination of mitochondrial dynamics proteins, such as Miro. In ALS neurons, the quality control mechanisms are affected by dysfunction occurring at various steps of the mitophagy process.

Mitochondrial dynamics, axonal transport, and MQC are tightly intertwined processes that play a fundamental role in the homeostasis of neuronal mitochondrial network, in health and disease. The genetics of familial ALS strongly suggest that these processes are affected during the course of the disease. Figure 1 summarizes the main molecular players of mitochondrial transport and turnover, whose alterations have been proposed to participate in ALS pathogenesis. More work is needed to achieve a detailed mechanistic understanding of the pathogenic pathways linking mitochondria transport and turnover with mitochondrial dysfunction and motor neuron degeneration in ALS. In particular, whether altered MQC in ALS motor neurons is the cause or the consequence of impaired mitochondrial function remains to be elucidated. Nevertheless, a wealth of clues indicate that alterations of these processes can be disease initiators or disease modifiers, with potentially interesting therapeutic implications. Studies focused on these important aspects of ALS pathophysiology will unveil novel disease mechanisms that could be addressed therapeutically by targeted approaches aimed at modulating mitochondrial transport and MQC through pharmacological intervention.
